# Verticillium Wilt Resistance in Solanaceous Crops: Genetic Resources, Grafting-Mediated Systemic Defense, and Implications for Productivity

**DOI:** 10.3390/plants15203206

**Published:** 2026-10-19

**Authors:** Fengqing Cheng, Zhigang Li, Yangyang Geng, Wenchao Du, Yang Cao

**Affiliations:** 1 College of Horticulture and Forestry, Tarim University/The National and Local Joint Engineering Laboratory of High Efficiency and Superior-Quality Cultivation and Fruit Deep Processing Technology of Characteristic Fruit Trees in Southern Xinjiang, Alar 843300, China; fengqingcheng1996@163.com (F.C.); 18609074519@163.com (Y.G.); 2 College of Horticulture, Hebei Agricultural University, Baoding 071000, China; 3 College of Biochemistry and Environmental Engineering, Baoding University, Baoding 071000, China; dwchge@163.com

**Keywords:** *V. dahliae*, V. albo-atrum, Solanaceae, Ve1, grafting, productivity, wild *Solanum*

## Abstract

*Verticillium* wilt, caused primarily by *Verticillium dahliae* and, in some hosts, *V. albo-atrum*, causes substantial yield losses in solanaceous crops. The disease is characterized by colonization of the vascular system, which can impair water transport and plant growth. Effective resistance therefore requires restricting vascular colonization while preserving vascular function and plant performance. Although considerable progress has been made in identifying resistance genes and defense responses, the molecular and physiological basis of *Verticillium* wilt resistance varies widely, and remains incompletely understood across solanaceous crops. Here, we summarize current knowledge of *Verticillium* wilt resistance in tomato, eggplant, potato, and pepper, focusing on vascular defense, immune signaling, lignification, and major resistance genes, loci, and quantitative genetic factors. We also discuss grafting-mediated resistance, highlighting resistant rootstocks and genetic resources, physiological and rhizosphere mechanisms of rootstock-mediated protection, and the potential roles of mobile signals in rootstock–scion communication. Furthermore, we examine the potential role of lignin remodeling as an interface between vascular defense and plant performance, while distinguishing disease-associated growth losses from potential defense-associated costs. We propose an integrative framework for evaluating resistance through pathogen restriction, vascular function, and agronomic performance, emphasizing that the relationships among these processes require further experimental validation. Finally, we outline future research priorities, including the identification and functional validation of resistance genes, quantitative assessment of vascular defense and its potential growth costs, and investigation of the causal contributions of grafting-associated signals and rhizosphere microbiomes. This review provides a framework for understanding *Verticillium* wilt resistance across solanaceous crops and highlights research priorities for developing durable resistance while maintaining crop productivity and quality.

## 1. Introduction

Solanaceous crops, including tomato (*Solanum lycopersicum*), eggplant (*S. melongena*), potato (*S. tuberosum*), and pepper (*Capsicum annuum*), are widely cultivated vegetable and food crops [1]. *Verticillium* wilt, caused primarily by *Verticillium dahliae* (*V. dahliae*) and, in some hosts, *Verticillium albo-atrum* (*V. albo-atrum*), is a destructive soilborne vascular disease that poses a substantial threat to their production [2]. *V. dahliae* is a hemibiotrophic ascomycete with a broad host range encompassing more than 400 plant species and produces microsclerotia that enable long-term survival in soil [3,4]. Disease management is difficult because microsclerotia can persist for years and no single cultural or chemical measure provides durable control across production systems [3,5]. Therefore, identifying resistance-related genes and understanding the molecular mechanisms underlying *Verticillium* wilt resistance are important for developing effective disease management strategies and accelerating resistance breeding.

The strength of mechanistic evidence differs among solanaceous crops. Tomato provides well-established functional evidence that the cell-surface leucine-rich repeat receptor-like protein (LRR-RLP) Ve1 recognizes the secreted *V. dahliae* effector Ave1 and activates downstream immune signaling [6,7]. However, reliance on a single race-specific resistance mechanism has limitations in practical applications, and its potential effects on crop productivity remain insufficiently evaluated. In eggplant, resistant wild relatives and related germplasm resources have been identified, and increasing attention has been directed toward the physiological and molecular mechanisms underlying their resistance [8,9,10,11,12,13,14,15,16]. Nevertheless, the translation of this extensive resistance diversity into commercial breeding and other practical applications remains limited. In potato, several resistance-associated loci have been reported, but their functional contributions and breeding utility remain incompletely resolved, while the early-dying complex further complicates interpretation of disease resistance [17,18,19,20,21,22,23,24]. Pepper remains comparatively understudied: resistant accessions and several vascular, metabolic, and transcriptional responses have been described, but their underlying genetic determinants are still poorly resolved [25,26,27,28,29,30]. Collectively, although diverse resistance germplasm and resistance-associated mechanisms have been identified across these solanaceous crops, their translation into genetically defined and practically deployable resistance for breeding remains limited. Bridging this gap between resistance discovery, mechanistic understanding, and breeding application is therefore a major challenge for improving *Verticillium* wilt resistance in solanaceous crops.

Grafting is one of the most widely used strategies for managing soilborne diseases in solanaceous crops, including *Verticillium* wilt. Although grafting-mediated resistance has traditionally been attributed largely to the physical and physiological barriers provided by resistant rootstocks, accumulating evidence indicates that grafting can also facilitate the movement of mobile RNAs, hormones, metabolites, and potentially epigenetic information, thereby potentially influencing scion responses to pathogen infection [31,32,33]. However, direct evidence linking these mobile signals to grafting-mediated resistance specifically against *Verticillium* wilt remains limited.

Current research on *Verticillium* wilt resistance has primarily focused on pathogen restriction and vascular defense, whereas the consequences of these defense responses for whole-plant physiological function, growth, and productivity have received comparatively limited attention. Although evidence from other plant–pathogen systems indicates that immune activation can affect plant growth and resource allocation [34,35,36], the magnitude and underlying mechanisms of potential growth–defense trade-offs have rarely been directly quantified in solanaceous–*Verticillium* interactions. These knowledge gaps highlight the need to evaluate resistance not only through pathogen restriction but also by considering vascular function and overall plant performance.

In this review, we summarize current knowledge of *Verticillium* wilt resistance across major solanaceous crops, focusing on genetic, physiological, and molecular resistance mechanisms, wild germplasm, and grafting-mediated systemic resistance. We further discuss potential relationships between vascular defense and plant performance. We therefore propose that effective resistance should be viewed as a dynamic balance among pathogen restriction, vascular protection, and resource allocation between defense and plant growth and reproduction. This review provides insights into the mechanisms underlying *Verticillium* wilt resistance and offers a foundation for developing sustainable resistance breeding strategies in solanaceous crops.

## 2. Vascular Colonization and Host Responses

**Figure 1:**
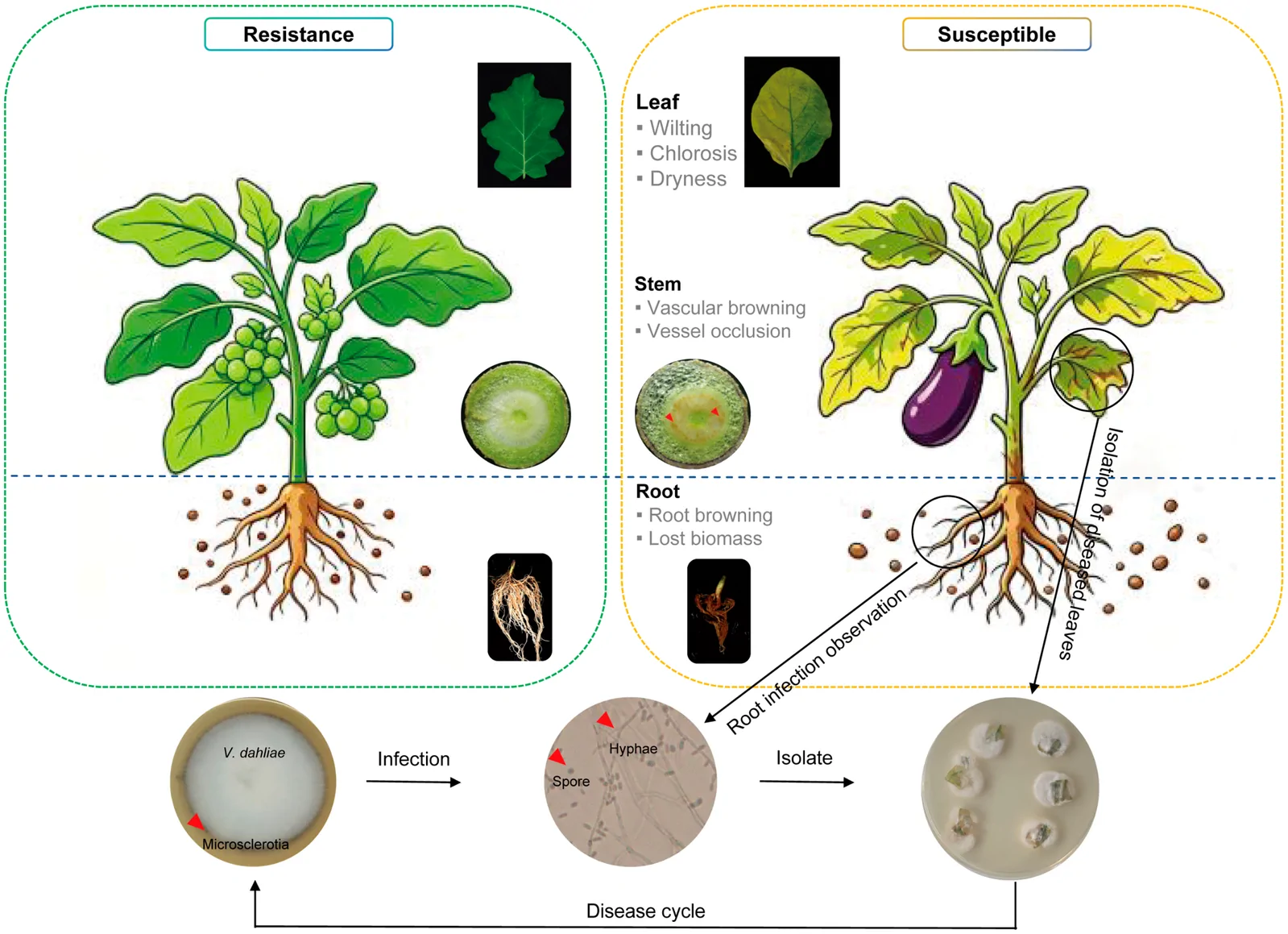
Infection cycle of *V. dahliae* and contrasting disease phenotypes of resistant *S. torvum* and susceptible cultivated eggplant (*S. melongena*). The infection cycle of *V. dahliae* is presented as a schematic illustration based on published knowledge, whereas the contrasting root, stem, and leaf phenotypes of resistant *S. torvum* and susceptible *S. melongena* represent experimental observations. Representative root, stem, and leaf phenotypes of susceptible *S. melongena* accession Sm01 and resistant *S. torvum* accession St01 were observed at 25 days post-inoculation (dpi) with *V. dahliae* strain VdGA. The plant and pathogen materials, inoculation procedures, and disease assessment methods were as previously described in the Materials and Methods section of the cited study [49].

*V. dahliae* causes *Verticillium* wilt in a wide range of plants, including major solanaceous crops such as eggplant, pepper, potato, and tomato, whereas *V. albo-atrum* has a relatively narrower host range and is mainly reported in tomato and potato [4]. Typical symptoms of *Verticillium* wilt in solanaceous crops include leaf chlorosis, wilting, and progressive growth suppression [3]. As a representative example, our own inoculation experiments showed that susceptible eggplant infected with *V. dahliae* developed pronounced root browning, vascular browning and vessel occlusion in stems, and progressive leaf chlorosis, wilting, necrosis, and desiccation, often accompanied by severe growth retardation. By contrast, resistant *S. torvum* exhibited no obvious disease symptoms in the roots, stems, or leaves under the same infection conditions (Figure 1). However, symptom development and disease severity vary depending on host genotype, pathogen virulence, and environmental conditions, resulting in differences among solanaceous crops and between resistant and susceptible genotypes [4].

The infection process of *V. dahliae* is dynamic and spatially coordinated, encompassing microsclerotial survival in soil, germination and growth toward host roots, root attachment and penetration, colonization of root tissues, xylem invasion and expansion, systemic vascular colonization, and symptom development [3,4]. The pathogen primarily survives in soil and plant residues as microsclerotia [37,38]. In response to signals from root exudates, microsclerotia germinate and produce hyphae that grow toward and attach to the root surface before penetrating the epidermis and colonizing root tissues [37,39,40]. Some hyphae attached to the root surface can further differentiate into specialized infection structures, such as appressoria and penetration pegs, which facilitate host penetration [41]. Following penetration of the epidermis and cortex, hyphae reach the vascular tissues and enter the xylem, marking a critical transition from localized root infection to systemic vascular colonization [42]. Within xylem vessels, the pathogen proliferates and produces spores that can be passively transported upward with the transpiration stream, thereby facilitating systemic colonization of stems and leaves [43]. Thus, the vascular tissues constitute a central interface at which pathogen proliferation and systemic spread are counteracted by host vascular defense responses. At later stages of infection, particularly as diseased tissues senesce, *V. dahliae* forms abundant microsclerotia within host tissues. These microsclerotia are subsequently released into the soil as infected residues decompose and can survive for extended periods, serving as a major source of primary inoculum for subsequent infections [42]. Collectively, these processes constitute the infection cycle of *V. dahliae* (Figure 1).

To restrict pathogen spread through the vascular system, plants activate a range of structural defenses, including lignification and suberization of vascular tissues and the formation of gels and tyloses that compartmentalize infected vessels and limit further fungal movement [43]. These structural responses are accompanied by biochemical defenses, including increased peroxidase activity, activation of the phenylpropanoid pathway, cell-wall reinforcement, and accumulation of antimicrobial secondary metabolites [44,45,46]. However, the effectiveness of these vascular defenses depends strongly on their timing, spatial distribution, and magnitude. Rapid and localized reinforcement may effectively restrict pathogen colonization, whereas delayed defense responses may be less effective, and excessive or widespread vessel occlusion can impair xylem conductivity and water transport [43,47]. Importantly, vascular blockage may also result from direct fungal colonization and infection-induced anatomical changes, rather than solely from host defense responses. For example, integrated analyses of *V. dahliae*-infected pepper showed that systemic fungal colonization was associated with substantial reductions in sap flow, water potential, and hydraulic conductance, with direct fungal blockage and anatomical alterations potentially contributing substantially to xylem dysfunction [48]. Thus, vascular defense should not be evaluated solely by the extent of structural reinforcement or vessel occlusion, but rather by whether these responses effectively restrict pathogen colonization while preserving xylem function. In this context, pathogen restriction primarily reflects resistance, whereas maintenance of vascular function and plant performance at a comparable pathogen burden may contribute to tolerance.

## 3. Resistance Sources and Molecular Mechanisms Across the Solanaceae

### 3.1. Tomato: Ve1 Paradigm of Verticillium Wilt Resistance

Tomato has served as one of the most extensively studied model crops for elucidating the genetic and molecular basis of resistance to *Verticillium* wilt. Genetic and map-based cloning studies demonstrated that resistance to race 1 of *V. dahliae* and *V. albo-atrum* is conferred by the *Ve* locus on chromosome 9, which contains two closely linked, inverted genes, *Ve1* and *Ve2*. Both genes encode cell-surface LRR-RLP proteins, with *Ve1* being the major determinant of race 1 resistance [6,50]. The cloning of *Ve1* provided a landmark example of RLP-mediated immune receptor and established an important framework for understanding *Verticillium* wilt resistance in plants.

The molecular basis of *Ve1*-mediated resistance was subsequently linked to recognition of the secreted effector Ave1 [7]. Ve1 protein lacks a cytoplasmic kinase domain, requires endoplasmic reticulum-resident chaperones for proper accumulation, and signals through the receptor kinase SOBIR1 [51,52]. Multiple immune components, including EDS1, NDR1, NRC1, ACIF, MEK2, and SERK3/BAK1, have also been implicated as positive regulators in *Ve1*-mediated immunity [53,54]. These findings identify required or contributing components, rather than a fully ordered linear cascade linking every component. Notably, activation of *Ve1* does not necessarily result in a classical hypersensitive response [55]. Instead, *Ve1*-associated resistance has been linked to rapid production of H_2_O_2_, enhanced peroxidase activity, activation of phenylalanine ammonia-lyase (PAL), and increased lignin deposition, suggesting that reinforcement of vascular tissues and restriction of pathogen movement constitute important defense outputs [50,56] (Table 1). Together, these responses indicate that reinforcement of vascular tissues and restriction of pathogen movement are important downstream outputs of *Ve1*-mediated immunity. *Ve2*, although insufficient to confer resistance independently, may contribute to the modulation of *Ve1*-mediated defense responses [57,58].

However, *Ve1*-mediated resistance is inherently race-specific because it depends on recognition of Ave1 [7]. Race 2 isolates that lack functional Ave1 evade *Ve1*-mediated recognition and can therefore overcome resistance conferred by the *Ve* locus [59]. Indeed, race 2 isolates were reported relatively soon after the deployment of *Ve*-carrying cultivars and subsequently became important in several tomato-growing regions [60]. A second layer of race-specific resistance has been identified in the tomato rootstock cultivars Aibou and Ganbarune-Karis, in which resistance to race 2 is controlled by the dominant *V2* locus introgressed from *Solanum neorickii* [61]. The corresponding fungal avirulence determinant, Av2, was subsequently identified in *V. dahliae*, and isolates capable of overcoming *V2*-mediated resistance are classified as race 3 [60,61]. The sequential emergence of the *Ve1*-Ave1 and *V2*-Av2 recognition systems provides a clear example of the dynamic interaction between host race-specific resistance and pathogen evolution, while also illustrating the vulnerability of resistance strategies that rely heavily on individual recognition specificities.

Importantly, the outcome of tomato–*V. dahliae* interactions cannot be explained solely by these defined race-specific resistance systems. Tomato can also exhibit substantial tolerance to non-adapted *V. dahliae* isolates [62], indicating that disease development is influenced by pathogen host adaptation and isolate-specific pathogenicity in addition to host resistance genes. Consequently, resistance observed in a particular tomato genotype or against a particular isolate cannot be assumed to provide equivalent protection across cultivars, pathogen populations, or production environments. Effective deployment of *Ve1*, *V2*, and other resistance loci therefore requires characterization of local pathogen populations, validation of resistance in recipient genetic backgrounds, and evaluation under relevant disease pressures. Moreover, direct quantitative evidence for the effects of *Ve1* itself on crop productivity remains limited. Future studies should therefore determine whether *Ve1*-mediated resistance influences plant growth, yield, or other agronomic traits under different genetic backgrounds and environmental conditions, thereby clarifying the relationship between race-specific immunity and crop performance.

### 3.2. Eggplant: Wild Germplasm as a Genetic and Mechanistic Resource

Cultivated eggplant is generally susceptible to *V. dahliae*, whereas resistance varies markedly among related wild *Solanum* species and accessions. Screening studies have identified accessions with relatively high levels of resistance in *S. aculeatissimum*, *S. scabrum*, and *S. sisymbriifolium* [63]. Resistance from *S. linnaeanum* has also been successfully introgressed into cultivated eggplant through interspecific hybridization and recurrent backcrossing [9]. In addition, eggplant materials carrying introgressed genomic segments from *S. aethiopicum* have exhibited increased resistance to *V. dahliae* [16]. Among these wild relatives, *S. torvum* has been particularly well studied as a source for dissecting the molecular basis of *Verticillium* wilt resistance [64,65,66]. Expression of *StoVe1* in transgenic potato increased resistance to *V. dahliae* [67]. *StoNPR1* overexpression and RNAi studies in potato support a role in resistance and SA-associated responses [10]. *StoLTPa7* encodes a lipid transfer protein induced by SA and *V. dahliae*; extracts from transgenic tobacco expressing this gene have shown antifungal activity [11]. *StoCYP77A2* expression in tobacco improved the disease response, and extracts from those plants inhibited fungal growth [12]. Silencing *StoWRKY6* and *StoMYB41* in *S. torvum* provided native-host functional support for their contribution to the tested disease response [13,14] (Table 1). Transcriptome–metabolome associations with phenylpropanoid, lignin, or phytoalexin pathways [15], identify hypotheses rather than a validated regulatory chain connecting all these genes. Importantly, resistance-related phenotypes observed following heterologous expression of these genes do not necessarily indicate equivalent effects in their native hosts or in commercially relevant genetic backgrounds. Further functional validation in the original species and agronomically useful breeding materials is therefore required to establish their contributions to resistance and potential breeding value.

These materials provide valuable resources for resistance-gene discovery and alien introgression. However, resistance can vary considerably among accessions and is influenced by pathogen isolates and environmental conditions. Therefore, resistance in wild eggplant relatives should be evaluated in the context of genotype × isolate × environment interactions rather than inferred solely from species identity [9]. This variability highlights the importance of assessing both disease symptoms and pathogen colonization to accurately characterize resistance. Moreover, translating these resistance resources into practical breeding applications remains challenging, particularly because interspecific introgression may be constrained by reproductive barriers and linkage drag.

In cultivated eggplant, immune receptor-associated signaling has also been implicated in defense against *V. dahliae*. BAK1, a well-established co-receptor involved in cell-surface immune signaling, was reported to participate in immune responses triggered by the *V. dahliae* effector VdPLP [68]. In addition, small RNAs contribute to the regulation of *Verticillium* wilt resistance. For example, miR395 has been reported to negatively regulate the response of eggplant to *V. dahliae* infection [69], whereas miRm0002 positively contributes to defense against *Verticillium* wilt [70]. These findings highlight post-transcriptional regulation as an additional layer of eggplant immunity.

Collectively, these studies highlight the value of wild eggplant relatives as sources of *Verticillium* wilt resistance and provide insights into the underlying molecular mechanisms. However, the genetic basis of resistance remains incompletely understood, and the utilization of wild resistance in cultivated eggplant is often constrained by reproductive barriers and linkage drag. Future studies should integrate resistance-gene discovery, functional validation, and breeding efforts to develop resistant cultivars and rootstocks with desirable agronomic traits.

### 3.3. Potato: Early Dying, Quantitative Resistance and Disease Tolerance

Potato early dying is a disease complex in which *V. dahliae*, *V. albo-atrum,* and the root-lesion nematode *Pratylenchus penetrans* can interact [17,18]. Nematode injury and changes in root function may favor fungal infection, and combined infection can produce disease outcomes that differ from either organism alone. Fungal species identification and nematode status should therefore be reported when comparing resistance.

Genetic studies further indicate that potato resistance to *Verticillium* wilt is controlled by multiple genomic regions whose effects may depend on population structure and developmental background. Mapping studies implicate *Ve*-related regions on chromosome 9 [19,20]. A diploid study identified a chromosome 5 QTL explaining approximately 48.2% of variation in its disease phenotype, overlapping *StCDF1*, together with a chromosome 9 locus near a *Ve2*-associated marker and epistasis between these regions [21] (Table 1). This population-specific estimate is not a universal measure of fungal restriction. Because *StCDF1* regulates maturity and tuberization, associations with canopy decline can reflect developmental timing as well as disease response. Co-localization does not prove that *StCDF1* is the causal resistance gene or that a *Ve2*-linked marker identifies a functionally validated receptor. In addition, a major segregating resistance determinant has been reported in *S. chacoense* [22], although its effectiveness requires further validation following introgression into agronomically useful genetic backgrounds.

At the transcriptional and metabolic levels, resistant potato genotypes may exhibit earlier or stronger activation of several defense-associated processes than susceptible genotypes. These responses include more rapid activation of SA-associated signaling, enhanced accumulation of defense-related specialized metabolites, and increased PAL activity, supporting an association between early immune activation, phenylpropanoid metabolism, and resistance [71]. However, such expression and metabolic differences alone do not establish the necessity or sufficiency of individual pathways for resistance. Similarly to the emerging evidence in eggplant, miRNAs have also been implicated in the regulation of potato responses to *V. dahliae* infection. For example, overexpression of miR482e increased susceptibility to *V. dahliae* [72], supporting a regulatory role for small RNAs in potato–*Verticillium* interactions, although the functional contributions of individual downstream targets require further validation.

A particularly important issue in potato is the distinction between resistance to pathogen colonization and tolerance to disease-associated yield loss. Because maturity strongly influences canopy senescence and PED symptoms, reduced symptom severity or delayed canopy decline does not necessarily indicate reduced fungal colonization [73,74,75]. Conversely, a tolerant genotype may sustain acceptable tuber yield despite substantial pathogen colonization. This distinction has both breeding and epidemiological implications: plants that maintain productivity while supporting fungal colonization may still contribute inoculum to subsequent crops. Therefore, evaluation of *Verticillium* resistance in potato should integrate disease symptoms, fungal colonization, maturity, and tuber yield under infected and mock-inoculated conditions. These complementary measurements would help distinguish resistance, characterized by reduced pathogen colonization, from tolerance, characterized by the maintenance of productivity despite infection.

### 3.4. Pepper: A Genetically Underexplored but Physiologically Diverse Defense System

Pepper remains one of the relatively understudied solanaceous hosts of *V. dahliae*. To date, no major race-specific resistance gene comparable to the tomato *Ve1* gene has been cloned and functionally characterized in pepper. Consequently, research on pepper *Verticillium* resistance has largely focused on resistant germplasm and physiological, biochemical, and transcriptomic responses associated with resistance. Nevertheless, systematic screening has revealed substantial genetic variation within the *Capsicum* germplasm pool. Among 397 wild and cultivated *Capsicum* accessions, several exhibited stable resistance to different *V. dahliae* isolates, although resistant commercial cultivars remained relatively uncommon [27]. This variation provides an important resource for dissecting the genetic basis of resistance and developing resistant pepper cultivars.

Physiological and transcriptomic evidence suggests that pepper resistance involves multiple defense processes, including phenylpropanoid metabolism, antioxidant defenses, sulfur metabolism, and cell-wall reinforcement. Resistant or tolerant cultivars can exhibit altered lignin composition, enhanced cell-wall cross-linking, sustained peroxidase activity, and transcriptional activation of defense pathways following *V. dahliae* infection [25,26,28]. The resistant cultivar ‘Yolo Wonder’ accumulates elemental sulfur (S^0^) earlier and more rapidly following infection, accompanied by changes in phenolic compounds, lignin deposition, and vascular tissues [29] (Table 1). These observations suggest that sulfur-associated responses may interact with phenylpropanoid metabolism and vascular defense, but the mechanistic and causal relationships among these processes remain unresolved. Most of these traits are supported by physiological or transcriptomic associations rather than direct functional evidence, and it remains unclear whether they represent causal determinants of resistance or downstream consequences of differences in pathogen colonization.

The host dependence of induced resistance further highlights the complexity of pepper defense. For example, the nonpathogenic *Fusarium oxysporum* strain Fo47 can protect pepper against *V. dahliae*, whereas comparable protection is not observed in tomato, despite Fo47 inducing defense-related responses in both hosts. Ethylene signaling contributes to the Fo47-induced response, but its role in disease resistance differs between pepper and tomato [30]. Collectively, these studies indicate that pepper possesses a physiologically diverse defense system involving metabolic reprogramming, redox regulation, sulfur-associated responses, vascular reinforcement, and hormone-mediated signaling. However, how these responses are genetically controlled, and which components are necessary for resistance, remain poorly understood.

This limited genetic resolution represents a major challenge for translating pepper resistance into breeding. In the absence of a functionally characterized major race-specific resistance gene, improvement currently relies largely on resistant germplasm and the selection of quantitative resistance traits [76]. Wild and related *Capsicum* taxa may provide additional resistance sources [27], but their utilization can be constrained by cross-incompatibility, reduced hybrid fertility, and linkage drag, including the co-introgression of undesirable alleles affecting fruit characteristics, pungency, flowering time, or yield. Moreover, if resistance is predominantly quantitative, marker-assisted selection must accommodate multiple loci whose effects may vary among genetic backgrounds and environments, making their deployment more complex than that of a single major resistance gene. Future priorities should therefore include standardized resistance screening using well-characterized *V. dahliae* isolates across environments, genetic mapping and validation of resistance loci in multiple backgrounds, and functional testing of candidate genes associated with cell-wall reinforcement, sulfur metabolism, and other defense processes. Importantly, these studies should integrate resistance evaluation with yield and fruit-quality measurements to determine whether enhanced disease resistance is accompanied by agronomic trade-offs.

**Table 1:** Reported *Verticillium* wilt resistance or tolerance determinants and sources across the Solanaceae.

Species	Determinant or Source	Pathogen Context	Background and System	Phenotype and Interpretation	Evidence and Limits
Tomato *(S. lycopersicum*)	*Ve1*	*V. dahliae* and *V. albo-atrum* race 1; Ave1-carrying isolates	Tomato and Arabidopsis genetic and receptor studies.	Cell-surface LRR-RLP that recognizes Ave1 and activates immune responses [6,7,50,56].	**Functional.** Strong receptor–effector evidence; resistance is Ave1-dependent and can be overcome by isolates lacking Ave1.
	*Ve2*	*V. dahliae* and *V. albo-atrum* race 1; Ave1-carrying isolates	Tomato and Arabidopsis genetic and receptor studies.	Associated with modulation of *Ve1*-mediated defense [6,57].	**Functional but dependent**. Does not independently confer *Ve1*-like resistance.
	Non-host-isolate response	*V. dahliae* tomato non-host isolate	Tomato infection and histology study.	Tolerance to a non-host *V. dahliae* isolate; reduced disease symptoms [62].	**Phenotypic**. No quantitative tolerance locus or causal determinant established by this study.
Tomato rootstocks (*S. neorickii*)	*V2*	*V.dahliae* race 2; Av2-carrying isolates	Resistant tomato rootstocks, including ‘Aibou’ and ‘Ganbarune-Karis’	Dominant resistance locus associated with recognition of Av2 [60,61].	**Genetic/functional**. Race-specific resistance; can be overcome by race 3.
Eggplant (*S. melongena*)	miR395	*V. dahliae*	Transgenic eggplant.	Overexpression increases susceptibility, supporting a negative role in resistance [69].	**Functional for disease phenotype.** Downstream mechanism requires further resolution.
	miRm0002	*V. dahliae*	Manipulation of miRNA activity in eggplant	Positive regulator of resistance; associated with activation of antioxidant defense [70].	**Functional for disease phenotype.** Predicted downstream targets require independent validation.
Pepper (*C. annuum*)	Phenylpropanoid- and lignin-associated defense	*V. dahliae*	Resistant/susceptible cultivar comparisons; physiological and lignin analyses.	Resistance/tolerance associated with lignin composition, cell-wall-bound phenolics, and maintenance of physiological performance [25,26].	**Physiological/correlative.** Supports pathway-level association; no individual causal determinant established.
*C. annuum*	Sulfur and expression candidates	*V. dahliae*	Comparative transcriptomic/physiological studies.	Differential sulfur accumulation and expression of defense-associated genes [28,29].	**Physiological/transcriptomic.** Candidate pathways rather than gene-level causal validation.
Potato (*S. tuberosum*)	*StVe1*-associated region	*V. dahliae*; *V. albo-atrum*	Tetraploid association; diploid/tetraploid linkage.	Genomic region/haplotype associated with reduced disease [19,20].	**Genetic association**. Does not by itself establish *StVe1* as the causal resistance gene.
	Chr5/Chr9 QTL; *StCDF1* overlap	*V. dahliae*	Diploid mapping population.	Disease-response QTL and epistasis [21].	**Genetic**. Maturity and developmental effects may confound apparent disease resistance.
	miR482e	*V. dahliae*	Transgenic potato.	Overexpression increased susceptibility [72].	**Functional for phenotype**. Contributions of individual downstream targets remain to be resolved.
*S. torvum*	*StoVe1*	*V. dahliae*; Ave1-associated recognition	Heterologous expression, including transgenic potato.	*Ve1* ortholog; enhanced *Verticillium* wilt resistance following heterologous expression; Ave1-related recognition reported [58,67].	**Functional,** partly heterologous. Native-host contribution and precise Ave1-dependent mechanism should be distinguished.
	*StoNPR1*	*V. dahliae*	Potato overexpression and RNAi.	Positive regulator associated with SA signaling [10].	**Functional in a heterologous system.** Native *S. torvum* requirement not established by this evidence.
	*StoLTPa7*	*V. dahliae*	Heterologous expression and antifungal assays.	Lipid transfer protein; antifungal activity [11].	**Biochemical/heterologous**. In vitro antifungal activity alone does not establish whole-plant resistance.
	*StoCYP77A2*	*V. dahliae*	Transgenic tobacco; antifungal assays.	CYP77-family protein associated with enhanced antifungal activity [12].	**Functional/heterologous.** Relevant downstream metabolites and native-host mechanism remain unresolved.
	*StoWRKY6*	*V. dahliae*	VIGS in *S. torvum.*	Positive contribution to defense responses [13].	**Functional in native host.** Downstream regulatory pathway remains incompletely resolved.
	*StoMYB41*	*V. dahliae*	VIGS in *S. torvum.*	Transcription factor associated with ABA-dependent defense regulation [14].	**Functional in native host.** Does not establish a complete ABA–MYB–resistance causal pathway.
*S. aethiopicum*	Introgression carrying *Rfo-Sa1*	*V. dahliae*	Introgressed eggplant material.	Expression and disease-related observations [16].	**Preliminary/associative.** Rfo-Sa1 is associated with *Fusarium* resistance; evidence presented here is insufficient to assign direct *Verticillium* resistance causality.
*S. linnaeanum*	Introgressed resistance source	*V. dahliae*	Interspecific introgression into eggplant.	Improved disease phenotype in introgressed material [9].	**Genetic/phenotypic.** Causal gene not identified; linkage drag and agronomic performance require evaluation.
*S. chacoense*	Major segregating determinant	*V. dahliae*	Wild-potato genetic segregation.	Major genetic component associated with high resistance [22].	**Genetic.** Causal gene and value after introgression into cultivated potato remain to be established.

## 4. Grafting Protection and Hypotheses for Systemic Signaling in Solanaceous Crops

### 4.1. Resistant Rootstocks and Genetic Resources

Grafting is widely used to manage soilborne diseases in solanaceous crops [77]. Wild relatives and interspecific hybrids represent important sources of genetic diversity for developing disease-resistant rootstocks, although many of these materials have been evaluated for resistance to soilborne diseases rather than specifically for *Verticillium* wilt. *S. torvum* is a widely used rootstock for eggplant, and experiments have reported reduced *Verticillium* wilt severity and improved growth or yield in particular graft combinations [65]. Other wild *Solanum* and *Capsicum* accessions remain primarily experimental germplasm or potential breeding resources. Moreover, their reported resistance to other soilborne pathogens should not be interpreted as direct evidence of resistance to *Verticillium* wilt. In tomato, resistant rootstocks are derived predominantly from wild tomato relatives, including *S. pimpinellifolium*, *S. habrochaites*, *S. chilense*, and *S. peruvianum*, which harbor diverse sources of resistance to soilborne pathogens and are frequently incorporated into interspecific or commercial rootstock breeding programs [78]. In eggplant, in addition to *S. torvum*, resistant rootstocks have been developed from *S. integrifolium*, *S. surattense*, and other wild *Solanum* species [79,80]. In pepper, related *Capsicum* species, including *C. chinense*, *C. frutescens*, and *C. baccatum*, provide genetic variation that may be useful for improving resistance to soilborne pathogens and environmental stresses [81]. Although not all of these rootstock resources have been specifically evaluated against *Verticillium* wilt, their potential resistance to soilborne pathogens warrant further evaluation for developing *Verticillium*-resistant rootstocks.

Disease resistance alone is insufficient to determine rootstock value. Successful rootstocks must combine resistance with graft compatibility, nursery uniformity, propagation efficiency, graft survival, appropriate vigor, earliness, fruit quality, abiotic-stress adaptation, and acceptable production costs [82]. Moreover, rootstock-mediated changes in water and nutrient acquisition can alter plant vigor and symptom expression independently of specific antifungal defense responses [83]. Environmental conditions, particularly temperature, humidity, and radiation in greenhouse production systems, can also influence disease development and the physiological performance of grafted plants [84]. Accordingly, we now discuss whether graft-mediated protection should be evaluated as an outcome of interactions among rootstock genotype, scion genotype, pathogen isolate, and production environment, rather than as a fixed property of a particular rootstock.

### 4.2. Physiological and Rhizosphere Mechanisms of Rootstock-Mediated Protection

The protective effects of resistant rootstocks may extend beyond the physical separation of susceptible scions from pathogen-infested soil [83]. Resistant rootstocks may influence plant defense through physiological responses, including reinforcement of root and vascular barriers, regulation of ROS homeostasis, and activation of phenylpropanoid metabolism. Evidence from resistant solanaceous genotypes provides support for the potential involvement of these processes. For example, enhanced peroxidase activity and lignin deposition are associated with vascular reinforcement, while antioxidant systems help limit oxidative damage during infection [15,56]. Together, these responses may contribute to restricting pathogen colonization while maintaining tissue function. However, much of this evidence derives from comparisons between resistant and susceptible genotypes rather than experiments specifically designed to demonstrate that resistant rootstocks induce similar responses in susceptible scions. Thus, although these physiological defenses may contribute to graft-mediated protection, their specific roles in systemic resistance remain to be established.

More direct evidence for rootstock-mediated effects has emerged at the root–soil interface. Grafting can alter root exudate composition and thereby modify the chemical environment surrounding roots. In eggplant, grafting has been shown to change both the composition and abundance of root exudates, with exudates from grafted plants exhibiting stronger inhibitory effects on *V. dahliae* mycelial growth than those from nongrafted plants [79]. GC–MS analyses further revealed substantial changes in the classes and relative abundances of root-exudate compounds following grafting, including hydrocarbons, esters, phenols, ketones, and other secondary metabolites [79]. Consistent with these observations, some compounds detected in grafted eggplant exudates, including dibutyl phthalate and fatty acids, have also been reported to inhibit *V. dahliae* growth in vitro [85]. Moreover, these exudate-associated metabolites may influence the broader rhizosphere microbial community. Several allelochemicals derived from grafted eggplant root exudates have been reported to suppress *V. dahliae* while increasing populations of antagonistic microorganisms in the rhizosphere [86]. Taken together, these findings suggest that rootstock-mediated changes in root exudates may influence pathogen activity through both direct chemical effects and indirect interactions with rhizosphere microorganisms. Nevertheless, the biological activities of individual compounds under field conditions and the causal relationships among root exudate composition, microbiome restructuring, and enhanced *Verticillium* wilt resistance remain insufficiently established.

### 4.3. Candidate Mobile Signals in Rootstock–Scion Communication

Grafting can modify the physiological and molecular state of the scion, potentially inducing or priming systemic defense responses [87]. These changes are often accompanied by transcriptional reprogramming involving defense-related pathways, hormone signaling, transcriptional regulation, and secondary metabolism. One possible mechanism underlying such systemic responses is the long-distance transport of signaling molecules, including hormones, RNAs, proteins, and metabolites, through the vascular system [33,88]. Among these candidates, mobile RNAs have received considerable attention because their movement across graft junctions has been experimentally demonstrated. Studies using Arabidopsis and *Nicotiana benthamiana*–tomato heterografts have shown that mRNAs move between scions and rootstocks, and some transcripts move bidirectionally between shoots and roots [32,89]. Mobile small RNAs can also move through the vascular system and regulate gene expression in recipient tissues [88]. Separately, interspecific grafting among tomato, eggplant, and pepper has been shown to alter DNA methylation patterns, with some changes persisting in sexually derived progeny [31]. However, these methylation changes should be interpreted as potential downstream consequences of grafting rather than evidence of mobile epigenetic signals.

Hormonal communication is another important component of rootstock–scion signaling. In tomato and eggplant, rootstock genotype can alter xylem-sap composition and hormone levels, including ABA and SA, potentially affecting water relations, vascular function, and stress responses in the scion [8,90]. Such hormonal changes could influence the physiological environment in which pathogen infection and host defense occur. However, altered hormone abundance in the scion does not necessarily indicate direct transport of these hormones from the rootstock, as it may also result from local biosynthesis, metabolic regulation, or differences in pathogen colonization [91,92].

Establishing a causal role for long-distance signals in graft-mediated resistance therefore requires demonstrating that a candidate signal originates in the rootstock, moves into the scion, and functionally contributes to disease protection. Rootstock-specific gene silencing, knockout, or overexpression combined with reciprocal and self-grafting could determine whether manipulating a candidate pathway specifically in the rootstock alters resistance in the scion. Graft-chimera or genotype-specific tracing approaches could distinguish rootstock-derived RNAs, proteins, or metabolites from molecules produced locally in the scion, while xylem-sap profiling and translocation assays could directly test the movement of candidate hormones and metabolites. For candidate mobile RNAs, demonstration of movement should be complemented by functional perturbation and analysis of downstream targets and disease phenotypes in the scion. These approaches should be combined with appropriate nongrafted and grafted controls, direct quantification of *V. dahliae* colonization, and relevant physiological measurements. Thus, although mobile signals provide a plausible basis for rootstock–scion communication in solanaceous crops, their causal roles in graft-mediated *Verticillium* wilt resistance remain to be established.

## 5. Vascular Defense and Productivity Evidence

### 5.1. Immune Signaling and Vascular Defense in Verticillium Wilt Resistance

**Figure 2:**
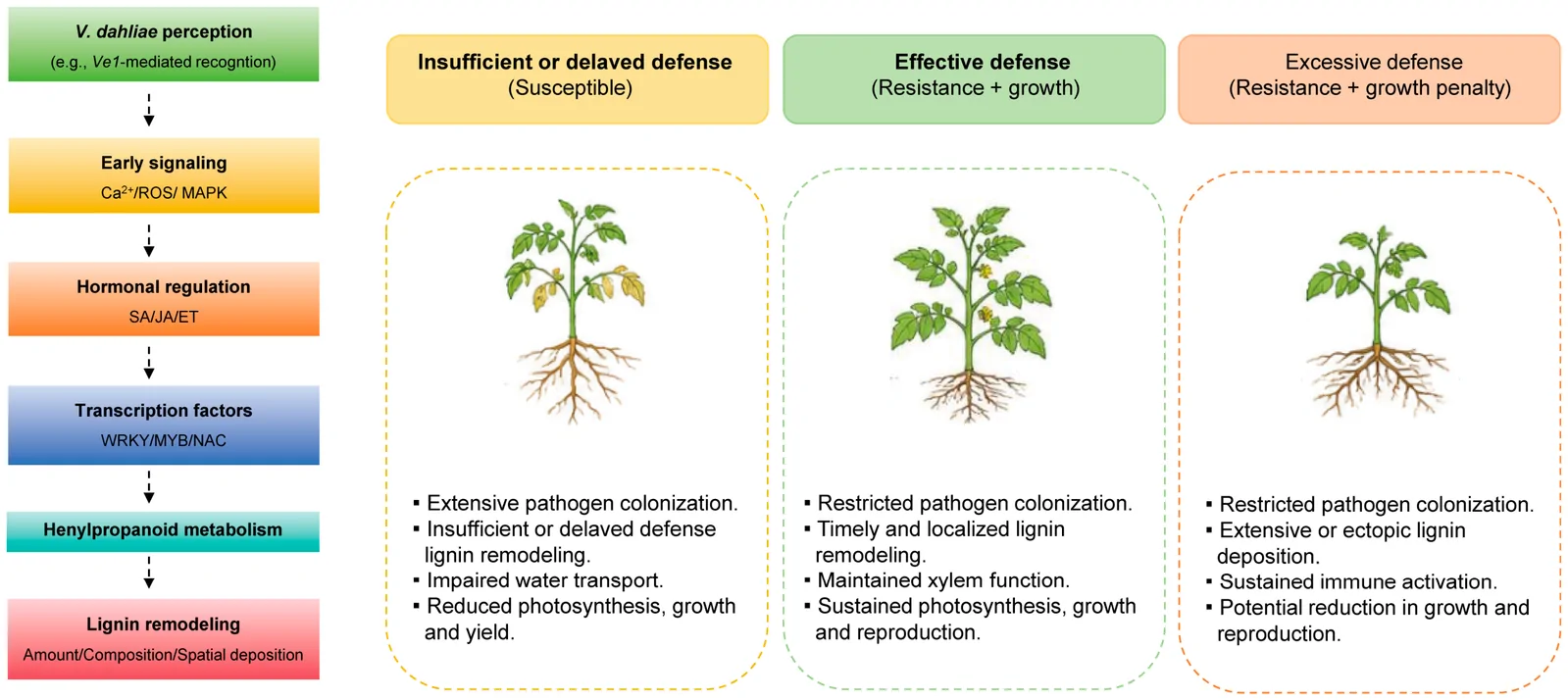
Conceptual framework linking vascular defense and plant performance during *V. dahliae* infection. This proposed model is based on evidence from different plant species and experimental systems and integrates established defense responses with hypothesized regulatory relationships. The proposed framework hypothesizes that pathogen perception activates interconnected signaling and transcriptional responses that promote phenylpropanoid metabolism and lignin remodeling, contributing to pathogen restriction and maintenance of vascular function. Insufficient defense can result in disease-associated losses, whereas excessive defense activation may impose potential defense-associated costs. The illustrated defense scenarios are hypothetical and are intended to provide a conceptual framework rather than quantitatively established response categories.

A useful working model connects perception, early signaling, hormone responses, transcriptional regulation, and vascular defense. However, the evidence comes from different hosts and experimental systems. Tomato Ve1 and SOBIR1 studies provide direct functional evidence for perception-associated immunity [7,50,52]. Studies of MAPK signaling and calcium sensors in cotton provide additional evidence for early defense signaling [93,94]. ROS may function as defense signals but can also accumulate as a consequence of pathogen-induced damage [95]. Regarding hormonal regulation, studies of SA-related responses in *S. torvum* and cotton [96], JA-mediated resistance in cotton [97], and ethylene (ET) signaling in Arabidopsis [98] provide evidence for the involvement of different hormone pathways, although their specific contributions may vary among host species. Furthermore, functional studies of WRKY and MYB transcription factors in *S. torvum* [13,14], together with a NAC expression survey in cotton [99], suggest the involvement of multiple transcriptional regulators. Together, these signaling and regulatory processes contribute to the activation of downstream defenses, including phenylpropanoid metabolism, lignification, and cell-wall reinforcement, which may help restrict fungal colonization within vascular tissues. However, individual components have largely been characterized in different species, genotypes, and experimental systems. Therefore, the proposed connections among early ROS/Ca^2+^/MAPK signaling, hormonal regulation, transcriptional reprogramming, and vascular reinforcement (Figure 2) should be viewed as an integrative conceptual framework rather than a single, experimentally established linear pathway.

Among these defense responses, phenylpropanoid metabolism and lignification have been repeatedly associated with resistance to vascular colonization. Classical studies of *Ve*-mediated resistance in tomato showed earlier H_2_O_2_ accumulation and peroxidase activation in resistant plants, followed by faster induction of PAL activity and greater lignin accumulation than in susceptible plants [56]. Similarly, in pepper, resistance or tolerance to *V. dahliae* has been associated with changes in lignin composition and increased accumulation of cell-wall-bound phenolics, which may contribute to cell-wall reinforcement and restriction of fungal colonization [17,18]. Consistent with these findings, resistant potato genotypes have also been reported to exhibit enhanced PAL activity and changes in phenylpropanoid metabolism in response to *V. dahliae* infection [72]. These observations suggest that cell-wall modification and lignin remodeling are important components of vascular defense across different solanaceous crops. Nevertheless, the extent to which these responses restrict pathogen colonization while preserving vascular function and plant growth remains insufficiently understood.

### 5.2. Lignin Remodeling as a Potential Interface Between Defense and Plant Performance

Lignin remodeling may represent an important functional interface between plant development and pathogen resistance. Lignin contributes to secondary cell-wall strength and vascular function, and defense-associated lignification can impede pathogen penetration and spread [100,101,102]. The potential relationship between lignification and plant performance is further illustrated by studies of lignin biosynthesis. Alterations in lignin biosynthesis can sometimes be accompanied by dwarfing, reduced biomass, or other developmental phenotypes, but these effects are strongly dependent on genetic background and the specific pathway component affected [100,101]. Moreover, such phenotypes cannot be explained simply by the diversion of carbon resources toward lignin production, as altered xylem function, cell-wall integrity signaling, and broader metabolic reprogramming may also contribute [103,104]. Thus, developmental changes associated with altered lignification do not necessarily demonstrate a direct metabolic trade-off between defense and growth. Likewise, lignin content alone is neither a simple proxy for defense intensity nor sufficient evidence for increased defense costs.

Taken together, these observations suggest that lignin remodeling may link pathogen restriction with vascular function and plant performance, although the nature of this relationship remains incompletely understood. We therefore consider propose lignin remodeling a potential functional interface between defense and growth, rather than as an established growth–defense trade-off node (Figure 2). In principle, effective vascular defense may depend on precise regulation of lignin deposition, allowing pathogen spread to be restricted while maintaining hydraulic function and minimizing negative effects on photosynthesis, biomass accumulation, reproduction, and yield. However, whether such coordination occurs across different solanaceous crops and infection conditions requires direct experimental validation.

### 5.3. Distinguishing Disease-Associated Costs from Potential Defense-Associated Costs

Insufficient or delayed defense can permit substantial disease-associated growth losses. When early immune responses fail to restrict *V. dahliae*, the pathogen can rapidly colonize xylem vessels and spread systemically, leading to vascular dysfunction, impaired water transport, leaf wilting, and ultimately severe reductions in plant growth and productivity [3]. These disease-associated losses arise primarily from pathogen colonization and the resulting physiological damage, rather than from the metabolic investment required for defense. Therefore, reduced growth in infected plants should not automatically be interpreted as evidence of a growth–defense trade-off.

Conversely, excessive or prolonged immune activation may also compromise plant performance, although through different mechanisms. Defense responses require metabolic resources, including carbon skeletons, ATP, reducing power, amino acids, nitrogen, and phenylpropanoid precursors. Under certain conditions, sustained immune signaling may alter resource allocation and metabolic regulation, potentially interfering with cell division, cell expansion, photosynthesis, and reproductive development [34]. Studies in crops further demonstrate that stronger immunity does not necessarily translate into superior agronomic performance, as some resistance genes or constitutively activated immune states can impose yield penalties [105]. However, these observations do not establish that enhanced resistance to *V. dahliae* necessarily incurs a growth penalty, particularly because much of the available evidence comes from other plant–pathogen systems. The classical *Ve1* resistance system in tomato provides a relevant context for considering the relationship between pathogen restriction and plant performance. Although *Ve1* is well established as a major determinant of race 1 resistance, the extent to which its protective effects are accompanied by the maintenance of physiological function and growth under disease pressure requires further evaluation [106]. Importantly, improved growth or yield in resistant plants under infection may reflect reduced disease damage rather than an additional growth-promoting function of the resistance gene itself [107]. Thus, the effectiveness of a resistance gene should be evaluated not only by its ability to restrict pathogen colonization but also by its contribution to maintaining plant performance under disease pressure.

Taken together, these observations highlight the need to distinguish two fundamentally different sources of growth loss in plant–pathogen interactions (Figure 2): disease-associated costs arising from insufficient pathogen restriction and potential defense-associated costs arising from the activation or maintenance of immune responses. The former primarily reflects pathogen-induced vascular dysfunction and its consequences for water transport, photosynthesis, and growth. The latter may involve metabolic investment, altered resource allocation, and regulatory interactions between defense and development [3]. Although such defense-associated costs have been documented in other plant systems, direct quantitative evidence in solanaceous–*Verticillium* interactions remains limited. Therefore, stronger defense should not be assumed to cause greater growth penalties, and any potential trade-off must be evaluated in relation to host genotype, pathogen pressure, environmental conditions, and the timing, intensity, and spatial distribution of defense responses.

Within this framework, effective resistance may depend less on maximal immune activation than on timely, localized, and coordinated defense responses that restrict vascular colonization while preserving plant physiological function. We therefore propose that *Verticillium* wilt resistance should be evaluated across three interrelated but independently measurable dimensions: pathogen restriction, vascular function, and plant performance (Figure 2). Rather than presuming a universal growth–defense trade-off, this framework provides a basis for determining whether, and under which conditions, defense activation is associated with measurable growth costs. Simultaneous measurements of pathogen burden, lignin content, composition and localization, hydraulic function, photosynthesis, biomass, reproductive output, and final yield within the same experimental system would help distinguish disease-associated losses from genuine defense-associated costs and identify defense strategies that maintain resistance without compromising agronomic performance.

## 6. Conclusions and Perspectives

Current evidence indicates that resistance to *Verticillium* wilt in solanaceous crops involves diverse genetic and physiological mechanisms, although the strength of experimental evidence varies considerably among species. Ve1–Ave1 recognition in tomato provides a well-established example of race-specific resistance. In eggplant, studies have emphasized the identification and utilization of resistant wild relatives and related germplasm resources, alongside the characterization of resistance-associated genes and defense mechanisms. In potato, QTL mapping has identified resistance-associated genomic regions, whereas studies in pepper have primarily revealed physiological and metabolic responses associated with resistance or tolerance. Despite these differences, several common defense-related processes have been reported across solanaceous crops, including immune signaling, oxidative stress regulation, phenylpropanoid metabolism, and cell-wall reinforcement. However, the genetic basis and strength of functional evidence differ among species. These findings suggest that solanaceous crops share certain defense-related responses but do not establish a single conserved causal network underlying *Verticillium* wilt resistance.

To advance mechanistic understanding and practical resistance improvement, three research questions should be prioritized. First, which resistance alleles or germplasm accessions confer stable resistance across different pathogen isolates and environmental conditions? Multi-isolate phenotyping, genetic mapping, and validation using near-isogenic or gene-edited lines should integrate pathogen quantification with disease symptoms. Second, which vascular defense responses effectively restrict pathogen spread while preserving hydraulic function, and under what conditions do they impose measurable growth costs? Comparative experiments involving infected and mock-inoculated plants should jointly assess lignin composition and localization, water relations, physiological performance, and productivity. Third, do rootstock-derived mobile signals or changes in rhizosphere microbial communities causally contribute to *Verticillium* wilt resistance? Demonstrating these relationships will require source-specific genetic perturbation, tracking of signal transport tracking, functional rescue experiments, and microbiome transfer or synthetic-community approaches, rather than relying solely on correlations between grafting, molecular changes, and disease suppression.

Translating these findings into crop improvement will require rigorous evaluation of resistance sources under genetically and environmentally diverse conditions. Resistance mechanisms identified in individual genotypes should be validated in commercial breeding backgrounds, while their applicability across species should not be assumed without additional functional evidence. For grafting-based strategies, rootstock selection should consider not only disease resistance but also graft compatibility, propagation costs, crop development, yield, and fruit quality. Breeding priorities should also reflect production systems. In low-technology systems, stable resistance across variable environments, local adaptation, and reduced management inputs may be particularly important, whereas highly controlled greenhouse systems may place greater emphasis on graft compatibility, rootstock–scion performance, yield stability, and fruit quality. Moreover, resistance effectiveness and durability should be assessed across pathogen isolates, growing seasons, soils or substrates, and climatic conditions, with attention to residual pathogen inoculum following harvest. Ultimately, the central breeding objective is to develop cultivars and grafting systems that provide durable disease protection while maintaining agronomic productivity and quality, an outcome that requires validation under practical production conditions.

## Data Availability

The data will be provided when it is requested.
